# A Funeral Oration

**Published:** 1891-05-02

**Authors:** 


### The Hospital

A Weekly Journal of

**Science, Medicine, Nursing, and Philanthropy.**

*For Hospitals, Asylums, Infirmaries, Dispensaries, Medical Practitioners, Students, Nurses, and the Charitable Public.*

Vol. X.—No. 240.] [May 2, 1891.

### A Funeral Oration.

Dead hypotheses sometimes receive the posthumous honours of funeral orations, as well as dead men. *De mortuis nil nisi bonum* rings in the ears and restrains the tongue of him who proclaims with subdued rhetoric the merits of deceased persons; but *de mortuis nil nisi malum* is, perhaps, more frequently the inspiring sentiment which lends energy to the language of him who denounces the demerits of a defunct hypothesis. Koch was promptly carried up to the stars by his medical and journalistic admirers less than six months ago. Unfortunately for him they left him at that elevation without continuous support; with the result that he has fallen into that miserablest of all Infernos, the Inferno of those who have persuaded learned and scientific persons to make themselves ridiculous. Koch's cure for tuberculosis is as dead—we will not say as Queen Anne—but as those Saxons who were killed at the Battle of Hastings, and whose gashed and dinted skulls may be seen to-day in the crypt of Hythe Church. Dr. Bristowe, F.R S., Senior Physician to St. Thomas's Hospital, is the English Antony who has been impelled by the strength of his convictions to pronounce the funeral oration on the dead Cæsar of Kochism.

In common with the rest of mankind, we cannot but feel profound regret that the new treatment of consumption, from which so much was expected, has proved in experience so entirely worthless. But we do not pretend to feel any regret at all that a method of treatment so demonstrably futile should be authoritatively laid to rest with appropriate burial rites. For our part, we had little hope and less faith in the Koch method from the very outset. On November 22nd, 1890, immediately after the publication of Koch's paper in the *Deutsche Medizinische Wochenschrift*, we wrote (The Hospital, November 22nd, 1890): "Three classes of patients who have been treated are mentioned: Firstly, those who were 'almost cured'; secondly, those in whom 'no improvement could be proved objectively'; and, thirdly, those who 'in the beginning can be cured.' We, along with millions of our fellow-creatures, wait for the addition to this list of a fourth class, viz., those who have been cured, definitely and permanently. Until this fourth class is added, and added not on the authority of Dr. Koch alone, or of his German confrères, but on the experience of competent physicians in other countries, we shall hold ourselves anxious to believe, but hard to be convinced." Two months later, when the general faith in Koch was still undiminished, we wrote (The Hospital, January 24th, 1891): "One fact of a very instructive character has emerged during the progress of the 'Koch Lymph' history. It is this: That scientific persons, even when considering and discussing scientific questions, are in all respects exceedingly like ordinary mortals. They follow leaders, they take sides, they rush to conclusions with unscientific haste, they make partial observations, they believe what they wish to believe rather than what the facts testify, and, having once taken up a position, they can hardly be compelled to move an inch, even at the bayonet's point of absolute and unquestionable demonstration. . . . So far we have considered but a small portion of Professor Virchow's judicial and admirably scientific paper. But we have quoted enough to justify two assertions, first that our original contention set forth in The Hospital immediately after the appearance of Koch's first paper, that the reaction caused by the injection of the lymph is not a reaction exclusively confined to tubercular tissue, but is, to a large extent, a general reaction, in which the tubercular tissues have no more than a share, is being confirmed; and secondly, that so far from the reaction being a tubercular reaction only, we may have a general reaction, so pronounced in particular parts as to kill the patient, in which, notwithstanding, the tubercular foci shall not be so much as touched. . . . The position which we have reached in the history of the Koch lymph is this, and no other—that a powerful fluid has been manufactured, and that it remains for sober practitioners to prove by a sufficient induction whether or not it will ever be of any value to mankind." How amply those early contentions have been since proved, doubters have only to read Dr. Bristowe's paper to clearly learn.

It is not necessary to say that Dr. Bristowe's conclusions rest upon the two indispensable bases of sound hypothesis and proved experience. Experiment alone is, of course, sufficient to convince all reasonable minds; but when it is shown not only that, in practice, a particular operation always fails to produce a particular result, but also that, from the very nature of the case, such an operation never can by any possibility produce such a result, and, further, that the operation both can and does bring about dangerous, and even fatal, consequences, then nothing is wanting to the absolute completeness of the demonstration. This is the feat which Dr. Bristowe has accomplished for the Koch treatment of consumption. It is impossible for us here even to outline the able and conclusive argument. Those who wish to study it will find the paper in the

*British Medical Journal* for April 25th. What we can do is to indicate some of Dr. Bristowe's more striking and convincing conclusions.

Dr. Bristowe points out that whilst vaccination and some other inoculations are purely protective proceedings, carried out in healthy persons, Koch's inoculation aims at the destruction of diseased tissues and at the cure of diseased organs. This is a fundamental distinction of the greatest possible importance. He also shows that though a specific bacillus is the cause of tuberculosis, Koch's treatment does not destroy the vitality of that bacillus or even affect it in any way. "So soon," he says, "as I learnt that Koch himself allowed that his injections had no directly injurious influence whatever upon the bacilli themselves, the essential cause of tuberculosis, the scepticism I had from the first entertained became at once profound; for I could not bring myself to believe that a mere aggravation of the natural inflammation that attends the tuberculous process, even if succeeded by extension of necrotic changes, could in any true sense lead to the cure of tuberculosis."

Good results, in the sense of cure, have not yet been obtained. But Dr. Bristowe points out with serious and urgent emphasis that evil consequences have had to be reckoned with. "No one," he says, "could contemplate with equanimity such aggravation of inflammation in the cases of tubercle of the lungs, bowels, brains, kidneys, or serous membrane, even though the temporary aggravation, failing to cause death, should lead to temporary improvement. In such cases as these, the only justification for such treatment seems to me to be the assurance of a reasonable hope that the temporary aggravation of mischief should be followed by an actual cure, and not merely by a lull in the progress of the malady."

It was claimed for the Koch lymph that though it did not destroy the tubercle bacilli, yet it specifically affected the tubercular tissues, and so checked the destructive process as to finally bring about a cure. Dr. Bristowe shows that this is a purely gratuitous assumption, irrational in theory, and destitute of proof. He believes that "any poisonous influence evoking high fever would tend to cause aggravation of inflammation in parts already the seats of inflammatory mischief, and that hence such effects might be expected to follow the introduction of any poison of the class to which Koch's fluid belongs; and, further, that had Koch's fluid been used as extensively in the treatment of the granulomata, such as syphilis and leprosy, as it has been in that of tubercle, one might have observed equivalent results." In other words, Dr. Bristowe believes two things, either of which is totally destructive of Koch's "specific" hypothesis. The first is that many substances of the septic class to which Koch's fluid belongs would produce exactly the same kind of reaction as that fluid; and the second that Koch's fluid itself, so far from acting on tubercular tissue alone, would, if tried, produce precisely similar effects upon syphilis, leprosy, and other granulomata. To sum up, Dr. Bristowe shows conclusively, both by reason and experience, that Koch's treatment fails to produce any single one of the good results claimed. It sets up no specific reaction; it effects no cure; it is often dangerous; it is sometimes fatal. Charity is permitted to hope that Koch's cure for consumption may not prove to be the winding-sheet of its discoverer's reputation.

